# Clinical features and outcomes of infective endocarditis in Egypt: an 11-year experience at a tertiary care facility

**DOI:** 10.1186/s43044-019-0018-y

**Published:** 2019-09-11

**Authors:** Hussein Hassan Rizk, Ahmed Adel Elamragy, Ghada Sayed Youssef, Marwa Sayed Meshaal, Ahmad Samir, Ahmed ElSharkawy, Karim Said, Hussien Heshmat Kassem, Mervat Gaber Elanany, Amani Ali El-Kholy, Al Sayed Akl, Soheir M. Mahfouz, Khaled Ali Sorour

**Affiliations:** 1 Department of Cardiology, Kasr Al Aini Hospital, Faculty of Medicine, Cairo University, Cairo, 11562 Egypt; 2 Department of Cardiothoracic Surgery, Kasr Al Aini Hospital, Faculty of Medicine, Cairo University, Cairo, 11562 Egypt; 3 Department of Clinical Pathology and Microbiology, Kasr Al Aini Hospital, Faculty of Medicine, Cairo University, Cairo, 11562 Egypt; 4 Department of Pathology, Kasr Al Aini Hospital, Faculty of Medicine, Cairo University, Cairo, 11562 Egypt

**Keywords:** Endocarditis, Registries, Egypt

## Abstract

**Background:**

Few data are available on the characteristics of infective endocarditis (IE) cases in Egypt. The aim of this work is to describe the characteristics and outcomes of IE patients and evaluate the temporal changes in IE diagnostic and therapeutic aspects over 11 years.

**Results:**

The IE registry included 398 patients referred to the Endocarditis Unit of a tertiary care facility with the diagnosis of possible or definite IE. Patients were recruited over two periods; period 1 (*n* = 237, 59.5%) from February 2005 to December 2011 and period 2 (*n* = 161, 40.5%) from January 2012 to September 2016. An electronic database was constructed to include information on patients’ clinical and microbiological characteristics as well as complications and mortality. The median age was 30 years and rheumatic valvular heart disease was the commonest underlying cardiac disease (34.7%). Healthcare-associated IE affected 185 patients (46.5%) and 275 patients (69.1%) had negative blood cultures. The most common complications were heart failure (*n* = 148, 37.2%), peripheral embolization (*n* = 133, 33.4%), and severe sepsis (*n* = 100, 25.1%). In-hospital mortality occurred in 108 patients (27.1%). Period 2 was characterized by a higher prevalence of injection drug use-associated IE (15.5% vs. 7.2%, *p* = 0.008), a higher staphylococcal IE (50.0% vs. 35.7%, *p* = 0.038), lower complications (31.1% vs. 45.1%, *p* = 0.005), and a lower in-hospital mortality (19.9% vs. 32.1%, *p* = 0.007).

**Conclusion:**

This Egyptian registry showed high rates of culture-negative IE, complications, and in-hospital mortality in a largely young population of patients. Improvements were noted in the rates of complications and mortality in the second half of the reporting period.

**Electronic supplementary material:**

The online version of this article (10.1186/s43044-019-0018-y) contains supplementary material, which is available to authorized users.

## Background

Infective endocarditis (IE) remains an evolving disease with high morbidity and mortality even in the modern era of advanced diagnostic imaging, improved antimicrobial chemotherapy, and potentially curative surgery [1]. Despite these improvements in diagnosis and management, the incidence of the disease is unchanged over the past two decades at approximately 1.7–6.2 cases per 100,000 per year [1] and has not appreciably changed recently. Almost all aspects of the disease, including its natural history, risk factors, sequelae, and causative organisms, have changed substantially since Osler’s first descriptions in the nineteenth century [2]. In particular, rheumatic heart disease (RHD) is now a less common risk factor, whereas degenerative valve disease in older individuals, mitral valve prolapse, intravenous drug use (IVDU), prosthetic valves, or devices have become the most common reasons for IE, coinciding with an increase in IE caused by staphylococcal infections and some fastidious organisms [1, 3]. Furthermore, pathogens that were previously difficult to detect and evolving multi-drug-resistant bacteria pose a challenge to the conventional therapeutic armamentarium [2]. These changes led to an alteration in IE epidemiology as well as outcomes over the past years. In Egypt, the prevalence of RHD is still high and the use of devices and prosthetic materials is also on the rise. This may put the population at high risk for IE. No national data on the IE burden is available, although it is imperative.

We reviewed all cases of IE referred to a tertiary care facility in Egypt and developed a registry with the goal of describing the clinical features and outcome of patients over a decade. A secondary goal was to describe the temporal trend in the disease outcome based on applying a bundle of interventions.

## Methods

### Study design and patient population

A specialized unit for IE was established in February 2005 in a tertiary care facility. It included cardiologists, cardiothoracic surgeons, laboratorians, radiologists, and pathologists. Data from all patients with possible/definite IE [4] was recorded in a specialized secure electronic database after thorough revision to build a registry which became part of two international multicenter registries: the International Collaboration on Endocarditis Prospective Cohort Study (ICE-PCS) in 2007 and the European Infective Endocarditis registry (Euro-ENDO registry) in August 2016.

This study involves all patients in the registry between February 2005 and September 2016. Patients’ data included demographics, clinical features, comorbid conditions, history, underlying heart disease, recent procedures, and history of IVDU. Early prosthetic valve endocarditis (PVE) was defined as IE that occurred within 1 year of surgical implantation of the prosthesis while IE that occurred afterwards was considered late PVE. Health-care associated IE (HAE) was defined as (1) nosocomial IE: IE occurred ≥ 48 h after hospital admission.(2) Non-nosocomial IE: IE occurring within (a) 1 month of receiving IV cannulation, chemotherapy, or dialysis; (b) 3 months after admission into an acute care facility; or (c) any time after admission into a nursing home. IVDU-associated IE was diagnosed if the patient reported injection drug use within 3 months of the onset of IE symptoms and was admitted from the community without an alternative risk factor for IE. Blood cultures, serologic tests for antibodies (against *Coxiella burnetii*, *Bartonella*, *Brucella*, and *Aspergillus*), surgical specimen cultures, and histopathologic examination were performed according to the IE unit protocol (Additional file 1). Transthoracic echocardiography (TTE) was performed within 24–48 h of hospital admission followed by transesophageal echocardiography within another 72 h if there was a clinical indication. Patients were followed for the duration of their hospital stay till either discharge or death, monitoring for in-hospital complications such as heart failure (HF), severe sepsis, intracranial hemorrhage, embolization, pulmonary infarcts, renal failure (defined as serum creatinine > 2 mg/dl), aortic root abscess, acute mitral or aortic regurgitation, splenic infarcts and abscesses, mycotic aneurysms, prosthesis-related complications (dehiscence, para-valvular leak, and stuck valve), and death. All patients underwent appropriate diagnostic modalities and received a full course of treatment with antimicrobials and surgical interventions as recommended by the current guidelines [5–11]. Prior to discharge, individual patient education was provided on need for IE prophylaxis, dental care, and symptoms and signs of IE recurrence. In addition to the regular patient care, the IE team in the specialized unit was involved in discussing complicated cases for decision-making, organizing seminars, and regular meetings to discuss clinical findings, preparing and distributing brochures about proper IE diagnosis, and management as well as arranging infection control awareness meetings and workshops on proper blood and tissue sampling techniques. Additionally, there were regular internal and external auditing meetings to monitor the progress of the IE registry.

### Statistical analysis

Analysis of data obtained from the database was done using SPSS 20.0 program. Data were expressed in terms of frequency as percentages for categorical variables. Continuous variables were presented as mean ± standard deviation (SD) if normally distributed or median and interquartile range (IQR) if skewed. Differences in categorical variables were tested by the chi-square test or Fisher’s exact (when appropriate). Comparison of means was tested by independent student *t* test. Significant univariate variables for in-hospital mortality were entered a multivariate binary logistic regression model to detect the most significant independent predictors. Statistical significance was defined as *p* values of < 0.05.

## Results

### Baseline characteristics

A total of 398 definite/possible consecutive IE patients were included in the IE registry between February 2005 and September 2016. We divided the registry into two periods according to the enrollment date. Period 1 (*n* = 237, 59.5%) included patients referred to the unit in the first 6 years (between 2005 and December 2011) and period 2 (*n* = 161, 40.5%) included patients referred between January 2012 and September 2016. The baseline clinical characteristics of the whole cohort and for each of the two periods are presented in Table 1.

**Table 1:** Baseline clinical characteristics

Variable	Total (*n* = 398), No. (%)	Phase 1 (*n* = 237), No. (%)	Phase 2 (*n* = 161), No. (%)	*P* value
Gender, male	243 (61.1)	139 (58.6)	104 (64.6)	0.232
Age, years	30 (24–39)^a^	29 (23–38)^a^	30 (24–40)^a^	0.414
Underlying cardiac diseases	255 (64.1)	191 (82.3)	64 (43.0)	< **0**.**001**
- Rheumatic heart disease	138 (34.7)	94 (40.5)	44 (29.5)	**0**.**029**
- Congenital heart disease	32 (8.0)	24 (10.3)	8 (5.4)	0.088
- Degenerative valve disease	10 (2.5)	7 (3.0)	3 (2.0)	0.550
- Floppy valves	12 (3.0)	8 (3.4)	4 (2.7)	0.463
- Hypertrophic cardiomyopathy	3 (0.8)	3 (1.3)	0 (0)	0.225
- Prosthetic valves IE	117 (29.4)	70 (29.5)	47 (29.2)	0.941
• AV mechanical prosthesis	58 (14.6)	32 (13.5)	26 (16.1)	
• AV bioprosthesis	5 (1.3)	3 (1.3)	2 (1.2)	
• MV mechanical prosthesis	60 (15.1)	39 (16.5)	21 (13.0)	
• MV bioprosthesis	1 (0.3)	0 (0)	1 (0.6)	
• TV bioprosthesis	2 (0.5)	0 (0)	2 (1.2)	
- Pacemaker/ICD^b^ lead IE	5 (1.3)	2 (0.8)	3 (1.9)	0.324
- Intravenous drug abuse	42 (10.6)	17 (7.2)	25 (15.5)	**0**.**008**
Healthcare associated IE	185 (46.5)	131 (55.3)	54 (33.5)	< **0**.**001**
* Hospital admission within the last 3 months	151 (37.9)	104 (43.9)	47 (29.2)	**0**.**01**
* Procedures within the last 3 months	95 (23.9)	71 (30.6)	24 (16.1)	**0.001**
- Intravenous line	37 (9.3)	20 (8.4)	17 (10.6)	0.475
- Early prosthetic valve IE^c^	27 (23.1)	20 (28.6)	7 (14.9)	0.69
- Hemodialysis	15 (3.8)	10 (4.2)	5 (3.1)	0.387
- Urinary catheter	2 (0.5)	1 (0.4)	1 (0.6)	0.646
- Non cardiac surgery	16 (4.0)	9 (3.6)	7 (4.3)	0.784
- Dental procedure	9 (2.3)	5 (2.1)	4 (2.5)	0.529
- Abortion/dilation and curettage	4 (1.0)	2 (0.8)	2 (1.2)	0.231
Fever	335 (84.2)	205 (86.5)	130 (80.7)	0.123
- Duration of fever, days	28 (13–60)^a^	21 (10–56)^a^	30 (14-60)^a^	**0**.**010**
Previous use of antibiotics	231 (58.0)	171 (72.2)	60 (37.3)	< **0**.**001**
Prior endocarditis	15 (3.8)	8 (3.4)	7 (4.3)	0.617

^a^Median, *IQR* interquartile range

^b^*ICD* implantable cardioverter defibrillator

^c^Percentages are related to the number of prosthetic valve IE

The patients were relatively young (median: 30 years, IQR: 24–39 years) and the majority were men. The main underlying cardiac disease was RHD. The percent of patients with history of IVDU was significantly higher in period 2 compared to period 1 while there was a significant decrease in those with RHD, HAE, and previous antibiotic use. Of the 185 patients with HAE, 76 (41.1%) patients had no underlying structural heart diseases [period 1: *n* = 47 (35.9%) vs. period 2: *n* = 29 (53.7%), *p* = 0.025]. The majority reported fever of long duration, which was significantly longer in period 2.

### Echocardiographic findings

The most pertinent echocardiographic findings are reported in Table 2. TTE was diagnostic in most of the patients (78.9%). Mitral valve was the most commonly affected valve (37.2%), followed by the aortic valve (28.6%). Patients from period 1 had more left-sided valvular involvement while right-sided valvular involvement was more common in period 2 as well as more ring abscesses. TTE was positive in significantly more patients in period 2.

**Table 2:** Echocardiography findings

Variable	Total (*n* = 398), No. (%)	Phase 1 (*n* = 237), No. (%)	Phase 2 (*n* = 161), No. (%)	*P* value
Diagnostic TTE	314 (78.9)	178 (75.1)	136 (84.5)	**0**.**025**
Presence of vegetations	298 (74.9)	175 (78.1)	123 (76.9)	0.772
- Left sided vegetations	240 (60.3)	154 (65.0)	86 (53.4)	**0**.**021**
• Aortic valve vegetations	114 (28.6)	73 (30.8)	41 (25.5)	< **0.001**
• Mitral valve vegetations	148 (37.2)	85 (49.0)	63 (39.2)	< **0.001**
- Right sided vegetations	68 (17.1)	23 (9.7)	45 (28.0)	< **0.001**
• Pulmonary valve vegetations	6 (1.5)	2 (0.8)	4 (2.5)	< **0.001**
• Tricuspid valve vegetations	62 (15.6)	20 (8.4)	42 (26.1)	< **0.001**
Ring abscess	52 (13.1)	25 (12.6)	27 (23.7)	**0**.**012**
Pericardial effusion	85 (21.4)	53 (27.2)	32 (24.1)	0.527
- Mild	73 (18.3)	46 (19.4)	27 (16.8)	
- Moderate	8 (2.0)	3 (1.3)	5 (3.1)	
- Large	4 (1.0)	4 (1.7)	0 (0)	

*TTE* transthoracic echocardiography

### Microbiologic data

Microbiological findings and antimicrobials used in treatment of IE are presented in Table 3. Patients from period 2 were significantly more likely to have negative blood cultures, but an organism was more likely to be detected in period 2. The most common organisms detected were staphylococcal species; with a significant increase in isolation of staphylococci from period 1 to 2, while there was a significant decrease in diagnosis of zoonotic IE. Fungal infections accounted for 8% of all cases. The demographic, clinical, and echocardiographic features of this subgroup were previously published [12].

**Table 3:** Microbiological and antimicrobial data

Variable	Total (*n* = 398), No. (%)	Phase 1 (*n* = 237), No. (%)	Phase 2 (*n* = 161), No. (%)	*P* value
Negative blood culture	275 (69.1)	152 (64.1)	123 (76.4)	**0**.**009**
Detected organisms^a^	155 (38.9)	83 (36.4)	72 (50.3)	**0**.**008**
- Staphylococcal species	88 (22.1)	46 (35.7)	42 (50.0)	**0**.**038**
- Streptococci	36 (9.0)	26 (20.2)	10 (11.9)	0.116
- Enterococci	16 (4.0)	9 (3.8)	7 (4.3)	0.991
- Gram-negative bacilli	32 (8.0)	22 (17.1)	10 (11.9)	0.304
- Zoonoses^b^	21 (5.3)	18 (14.0)	3 (3.6)	**0**.**009**
- Fungal infection^c^	32 (8.0)	17 (7.2)	15 (9.3)	0.440
Basis of choice of antibiotics				
- Culture/Serology based	86 (21.6)	42 (17.7)	44 (27.3)	**0**.**022**
- Empirical	312 (78.4)	195 (82.3)	117 (72.7)	
Therapeutic antibiotics used^d^	(*n* = 1044)	(*n* = 610)	(*n* = 434)	
- Anti-Staphylococcal^e^	228 (21.8)	120 (19.7)	108 (24.9)	**0**.**045**
- Aminoglycosides	310 (29.7)	162 (26.6)	148 (34.1)	**0**.**009**
- Quinolones	20 (1.9)	3 (0.5)	17 (3.9)	< **0.001**
- B-lactam and Cephalosporins	338 (32.4)	233 (38.2)	105 (24.2)	< **0**.**001**

^a^Organisms are detected by either blood culture, positive histology or positive serology

^b^Zoonotic infections included: Brucella, Bartonella, and Coxiella

^c^Fungal infections included: Aspergillus, Candida, Mucormycosis, and Penicillium

^d^Numbers are calculated according to the total number of antibiotics used

^e^Anti-Staphylococci included; Vancomycin, Linezolid, and Teicoplanin

### Complications

Complications and outcomes are shown in Table 4. The overall complication rate (especially HF) decreased significantly from period 1 to 2 while the diagnosis of splenic and pulmonary infarcts increased significantly. Response to medical management was noted in approximately 47%, with surgical indication in 74% of patients overall and in-hospital mortality of 27%. There was a significant improvement in response to medical management and a significant decrease in in-hospital mortality noted between period 1 and 2.

**Table 4:** Complications of IE

Variable	Total (*n* = 398), No. (%)	Phase 1 (*n* = 237), No. (%)	Phase 2 (*n* = 161), No. (%)	*P* value
Overall complications	157 (39.4)	107 (45.1)	50 (31.1)	**0**.**005**
- Heart failure	148 (37.2)	98 (41.4)	50 (31.1)	**0**.**011**
- Severe sepsis	100 (25.1)	56 (23.6)	44 (27.3)	0.304
- Renal failure (Creatinine > 2 mg/dl)	82 (20.6)	48 (20.2)	34 (21.1)	0.941
• Need for dialysis	18 (4.5)	13 (5.5)	5 (3.1)	0.236
- Acute mitral regurgitation	37 (9.3)	21 (8.9)	16 (9.9)	0.779
- Papillary muscle rupture	4 (1.0)	2 (0.8)	2 (1.2)	0.545
- Acute aortic regurgitation	26 (6.5)	14 (5.9)	12 (7.5)	0.585
- Splenic infarction	38 (9.5)	18 (7.6)	20 (12.4)	0.123
- Splenic abscess	19 (4.8)	7 (3.0)	12 (7.5)	**0**.**044**
- Pulmonary infarction	31 (7.8)	10 (4.2)	21 (13.0)	**0**.**001**
- Prosthesis-related complications	33 (8.3)	21 (8.9)	12 (7.5)	0.709
• Dehiscence	17 (4.3)	12 (5.1)	5 (3.1)	
• Para-valvular leak	10 (2.5)	5 (2.1)	5 (3.1)	
• Stuck valve	6 (1.5)	4 (1.7)	2 (1.2)	
- Mycotic aneurysm	24 (6.0)	11 (4.6)	13 (8.1)	0.167
- Intracranial hemorrhage	26 (6.5)	17 (7.4)	9 (5.6)	0.481
- Peripheral embolization	133 (33.4)	78 (32.9)	55 (34.2)	0.941
• CNS embolization	50 (12.6)	34 (14.3)	16 (9.9)	0.152
Outcomes				
- Response to medical treatment	185 (46.5)	90 (38.0)	95 (59.0)	< **0**.**001**
- Indication for surgery	294 (73.9)	180 (75.9)	114 (70.8)	0.252
• Surgery performed	176 (59.9)	113 (62.8)	63 (55.3)	0.092
- In-hospital mortality	108 (27.1)	76 (32.1)	32 (19.9)	**0**.**007**

### Predictors of in-hospital mortality

In univariate analysis, predictors included early prosthetic IE, overall complication rate, severe sepsis, intracranial hemorrhage, lack of response to medical therapy, presence of surgical indication, not performing indicated surgery, HAE, and pericardial effusion. A multivariate logistic regression model—that correctly classified 89% of cases—determined the most significant independent factors associated with increased risk of in-hospital mortality: (1) severe sepsis (OR 23.6, 95% CI 9.6–58.3, *p* < 0.001), (2) intracranial hemorrhage (OR 3.5, 95% CI 1.5–7.7, *p*: 0.003), (3) any complications (OR 3.4, 95% CI 2.2–5.4, *p* < 0.001), (4) early prosthetic valve IE (OR 3.2, 95% CI 1.4–7.0, *p*: 0.003), (5) any indication for surgery (OR 3.0, 95% CI 1.6–5.6, *p* < 0.001), (6) HAE (OR 2.0, 95% CI 1.3–3.2, *p*: 0.003), and (7) underlying heart disease (OR 1.8, 95% CI 1.1–2.9, *p*: 0.02).

Factors associated with a decreased in-hospital mortality included (1) response to medical treatment (OR 0.03, 95% CI 0.01–0.08, *p* < 0.001), (2) zoonotic infection (OR 0.3, 95% CI 0.1–1.2, *p*: 0.05), and (3) performing indicated surgery (OR 0.4, 95% CI 0.3–0.7, *p* < 0.001).

## Discussion

This is the first published comprehensive report from Egypt on all patients with IE admitted to a tertiary care facility over 11 years. Reports of subgroup analysis were previously published elsewhere [12–18]. In this cohort, we observed certain characteristics that may be particularly relevant to other low-resource countries.

The patient population was relatively young (median 30 years), similar to reports from Lebanon [19] and Saudi Arabia [20] but in contrast to other registries from resource-rich countries such as Italy [21] (median 57 years) and China [22] (mean 43.5 years). This difference is likely due to the difference in underlying heart disease. In Egypt (as in many other low-resource countries), RHD remains a common underlying heart disease followed by prosthetic valves (usually indicated for RHD) while in the resource-rich countries, degenerative heart diseases of the elderly, and increasing use of prosthetic devices (valves and pacemakers) are the main underlying etiology.

Another striking feature from our data is the long duration of symptoms (i.e., fever) prior to admission with the diagnosis of IE (median 28 days). This cohort includes patients referred to a tertiary care facility receiving patients from rural and other under-served areas in Egypt. This may account for the long duration of prior symptoms. The usual symptoms and signs associated with IE are non-specific. Thus, unless the clinician maintains a high index of suspicion, diagnosis can be missed. This long duration of symptoms prior to diagnosis may also have resulted in the high rates of complications and adverse outcomes. In a study of IE conducted in France [23], 25% of patients were diagnosed late (> 1 month after onset of symptoms) and this delay was associated with higher rates of surgical management.

HAE accounted for 46.5% of cases in this registry; 95 (51.4%) of them had a history of predisposing procedure in the preceding 3 months. The most common was valve replacement surgery with associated early PVE (28.4%) followed by intravenous lines (20%). These findings are consistent with the 34% reported by ICE-PCS [24] and with the global increase in medical procedures, devices and health care services.

The rate of culture-negative IE was high among our patients which complicates the ability to make definitive diagnosis. This may be attributed to the use of antibiotics for treatment of antecedent febrile illness prior to hospital admission and the drawing of blood cultures. In literature, the rate of culture-negative IE ranges between 2.5 and 31% [25–28]. This rate was high in Algeria [29] (56.4%) and South Africa [30] (55.3%) compared to the UK [31] (12.2%) and France [32] (9%).

### Complications

The overall complication rate was high (39.4%), similar to other reports [19]. The main complications were potentially life-threatening, including HF, severe sepsis, and renal failure, which likely contributed to the high in-hospital mortality. Late diagnosis and the high incidence of the Staphylococcal species are contributing factors, in addition to the inability to perform surgery in some patients when indicated. A similar short-term mortality result (20%) was reported in a previous meta-analysis [33]. Predictors of mortality have varied in different studies. In one study [34], the main predictors of higher mortality were chronic obstructive pulmonary disease, cerebral embolism, Staphylococcus, and gram-negative bacilli as well as surgical treatment when indicated. In a nation-wide survey in Japan [35], the main predictors of higher mortality were *Staphylococcus aureus* infection and HF while early surgery was associated with a decrease in mortality.

### Temporal trends

There were significant changes noted over the period of assessment. Comparing the findings from period 1 (the first 6 years) and period 2 (the following 5 years) showed a significant decrease in RHD as an underlying disease for IE with an increase in IVDU-associated IE, with predominance of Staphylococcal species as a causative organism. HAE and prior antibiotic use decreased significantly in period 2. However, the median duration of symptoms prior to diagnosis increased, highlighting the need for provider and patient education regarding risk factors, symptoms and signs of IE, and the importance of early diagnosis.

During the period of observation, there was also a substantial change in the types of causative organisms. Staphylococcal IE increased significantly in period 2 due to increased IVDU; and although the percent of IE cases with negative cultures also increased in the same period, identification of the causative organism improved significantly owing to the use of more sophisticated diagnostic methods like serologic tests and polymerase chain reaction (PCR) of histologic specimens. While these techniques were used routinely in period 2, they were restricted in period 1 to situations of clinical suspicion of zoonotic infection in the presence of negative blood cultures. Similar findings were reported in a study conducted in Algeria [29] where the application of serologic tests and PCR techniques decreased the proportion of IE patients with unidentified organisms from 56.4 to 30.9%; while in France [32], this rate dropped from 9 to 5%. Thus, these techniques are important tools to enhance pathogen identification. However, these techniques are expensive and not readily available in all clinical settings, limiting their routine use in the country.

### Limitations

This study was conducted at a tertiary care facility involving high numbers of referred patients and with the availability of sophisticated diagnostic and therapeutic capabilities [36–42], leading to selection and referral bias, and creating difficulty in generalizability of the findings [43–45]. Population-based studies—that may provide better information—are few and have their own limitations as well [46]. Thus, our study as well as similar studies from tertiary care centers likely includes severe and complicated cases. In addition, the more developed diagnostic and therapeutic capabilities at our center may also have favorably impacted the outcomes of the patients.

## Conclusions

This study is the first to describe the clinical features, microbiological characteristics, and outcome of patients with IE admitted to a tertiary care facility in Egypt over 11 years. High rates of culture-negative IE, IVDU, complications, and mortality as well as the young age of the patients and long duration of symptoms prior to diagnosis were the most prominent features.

Over the period of observation, there were improvements noted in the detection of the causative organisms and in outcomes, including rates of complications and mortality in the second period.

Despite this progress, IE remains a serious and deadly disease. A third of patients experienced a serious complication, and a fifth died during their hospitalization. Further efforts are needed to disseminate information regarding the risk factors for IE, to educate providers on early diagnosis and to continue to expand access to preventive, diagnostic, and therapeutic interventions in the country.

## Additional file

Additional file 1: Microbiology methods. (DOCX 33 kb)

## Data Availability

The datasets used and/or analyzed during the current study are available from the corresponding author on reasonable request.

## Abbreviations

Euro-ENDO registry: European Infective Endocarditis registry
HAE: Health-care associated IE
HF: Heart failure
ICD: Implantable cardioverter defibrillator
ICE-PCS: International Collaboration on Endocarditis Prospective Cohort Study
IE: Infective endocarditis
IQR: Interquartile range
IVDU: Intravenous drug use
PCR: Polymerase chain reaction
PVE: Prosthetic valve endocarditis
RHD: Rheumatic heart disease
SD: Standard deviation
TTE: Transthoracic echocardiography
