# Supplementary material for: Clinical features and outcomes of infective endocarditis in Egypt: an 11-year experience at a tertiary care facility
Source: Egypt Heart J. 2019 Sep 11;71:17. doi: 10.1186/s43044-019-0018-y (PMC6821432; doi:10.1186/s43044-019-0018-y)
Supplement: Supplementary file 1 — Microbiology methods. (DOCX 33 kb) [file 43044_2019_18_MOESM1_ESM.docx]

**Additional file**

Microbiology methods:

All patients were subjected to blood sampling for blood culture and serodiagnosis, while those who were candidates for surgical valve replacement, were subjected to valve analysis as well. Laboratory procedures were done according to standard methods (1-3).

1. Blood cultures sampling: From each patient, at least 3 sets of blood culture were collected with the first and last samples drawn at least 1 hour apart; from separate venepunctures before starting antibiotics. Each set consisted of one BACTEC Plus aerobic/F and one BACTEC Plus anaerobic/F blood culture vials (Becton, Dickinson Sparks, MD, UAE). Around 10ml/bottle were aseptically withdrawn from adults. For paediatric patients, the volume was according to weight. Blood culture bottles were incubated in BACTEC 9240 instrument for 14 days. Positive blood culture bottles were subcultured on 5% Sheep blood agar, chocolate blood agar, MacConkey agar and Sabouraud dextrose agar plates (Oxoid Ltd, UK). Microbial colonies were identified by Gram stain, colony morphology and by VITEK-2 (BioMerieux)[1]^.^
2. Surgically excised materials including excised valves, vegetations, infected prosthesis, aortic abscess and emboli were submitted to the microbiology laboratory for Gram stain, potassium hydroxide preparation (KOH) and bacterial and fungal cultures on 5% Sheep blood agar, chocolate blood agar, MacConkey agar and Sabouraud dextrose agar plates (Oxoid Ltd, UK), and for histopathology examination. Organism identification and susceptibility testing were done by VITEK-2 (BioMerieux)[2]^.^
3. Serodiagnosis: this was done for detection of antibodies to endemic zoonotic agents (Brucella, Bartonella and Coxiella) and Aspergillus Galactomann antigen according to manufacturer instructions. Anti-Brucella antibodies were detected using the tube agglutination test (Linear Chemicals, Montgat-Barcelona, Spain). Testing for IgG antibodies against Bartonella henselae and Bartonella quintana and for IgG, IgM, and IgA antibodies against Coxiella burnetii was carried out using the indirect immunofluorescence assay (Vircell S.L. microbiologist, Granada, Spain). A patient was considered to have brucellosis when antibody titers for Brucella were at least 1/320, Bartonella endocarditis when IgG titers were at least 1: 800, and Coxiella endocarditis when phase I IgG titre was at least 1: 800. Aspergillus galactomannan antigen detection was carried out using the Platelia EIA (BioRad). Though patients with an index > 0.5 were considered positive for Aspergillus antigen according to the kit, yet we used an index >1 for diagnosis of Aspergillus endocarditis, based on the more than double fold higher index values observed in patients with positive cultures for Aspergillus[3].

**References**

[1] J.S. Li, D.J. Sexton, N. Mick, R. Nettles, V.G. Fowler, T. Ryan, T. Bashore, G.R. Corey, Proposed Modifications to the Duke Criteria for the Diagnosis of Infective Endocarditis, Clin. Infect. Dis. 30 (2000) 633–638. doi:10.1086/313753.

[2] J.H. Jorgensen, Manual of Clinical Microbiology, 11th Edition, 11th ed., American Society of Microbiology, Washington, DC, 2015. doi:10.1128/9781555817381.

[3] L.M. Baddour, W.R. Wilson, A.S. Bayer, V.G. Fowler, I.M. Tleyjeh, M.J. Rybak, B. Barsic, P.B. Lockhart, M.H. Gewitz, M.E. Levison, A.F. Bolger, J.M. Steckelberg, R.S. Baltimore, A.M. Fink, P. O’Gara, K.A. Taubert, Infective Endocarditis in Adults: Diagnosis, Antimicrobial Therapy, and Management of Complications, Circulation. 132 (2015) 1435–1486. doi:10.1161/CIR.0000000000000296.
